# Supplementary material for: Epidemiological Analysis of Suicidal Behaviour in Spain from 2017 to 2022 and Comparative Perspectives with Japan: A Retrospective Observational Study
Source: Healthcare (Basel). 2025 Feb 20;13(5):451. doi: 10.3390/healthcare13050451 (PMC11899283; doi:10.3390/healthcare13050451)
Supplement: Supplementary file 1 [file healthcare-13-00451-s001.zip › healthcare-3431031-supplementary.pdf]

STROBE Statement—Checklist of items that should be included.

	Item No	Recommendation	Location where item is reported
**Title and abstract**	1	(*a*) Indicate the study's design with a commonly used term in the title or the abstract	In title and in the "Methods" section in the abstract.
		(*b*) Provide in the abstract an informative and balanced summary of what was done and what was found	In the sections "Methods" and "Results" of the abstract.
**Introduction**			
Background/rationale	2	Explain the scientific background and rationale for the investigation being reported	From subsections 1.1. to 1.4. background is explained, and in subsection 1.5. the rationale of the study.
Objectives	3	State specific objectives, including any prespecified hypotheses	The objectives are in subsection 1.5. (lines 133-137) and the research questions are in subsection 1.6.
**Methods**			
Study design	4	Present key elements of study design early in the paper	In the subsection 2.1. Study design and setting.
Setting	5	Describe the setting, locations, and relevant dates, including periods of recruitment, exposure, follow-up, and data collection	In the subsection 2.1. Study design and setting.
Participants	6	(*a*) Give the eligibility criteria, and the sources and methods of selection of participants. Describe methods of follow-up	In the subsection 2.1. Study design and setting.
		(*b*) For matched studies, give matching criteria and number of exposed and unexposed	Not applicable
Variables	7	Clearly define all outcomes, exposures, predictors, potential confounders, and effect modifiers. Give diagnostic criteria, if applicable	In the subsection 2.2. Variables and data sources.
Data sources/ measurement	8*	For each variable of interest, give sources of data and details of methods of assessment (measurement). Describe comparability of assessment methods if there is more than one group	In the subsection 2.2. Variables and data sources.
Bias	9	Describe any efforts to address potential sources of bias	Bias is explained on subsection 2.4. Risk of bias.
Study size	10	Explain how the study size was arrived at	In the subsection 2.2. Variables and data sources.
Quantitative variables	11	Explain how quantitative variables were handled in the analyses. If applicable, describe which groupings were chosen and why	In the subsection 2.2. Variables and data sources.
Statistical methods	12	(*a*) Describe all statistical methods, including those used to control for confounding	In the subsection 2.3. Statistical analysis.
		(*b*) Describe any methods used to examine subgroups and interactions	In the subsection 2.3. Statistical analysis.
		(*c*) Explain how missing data were addressed	Not applicable

		(*d*) If applicable, explain how loss to follow-up was addressed	Not applicable
		(*e*) Describe any sensitivity analyses	Not applicable
**Results**			
Participants	13*	(a) Report numbers of individuals at each stage of study—eg numbers potentially eligible, examined for eligibility, confirmed eligible, included in the study, completing follow-up, and analysed	In the subsection 3.1. Sample description.
		(b) Give reasons for non-participation at each stage	Not applicable
		(c) Consider use of a flow diagram	Not applicable
Descriptive data	14*	(a) Give characteristics of study participants (eg demographic, clinical, social) and information on exposures and potential confounders	In the subsection 3.1. Sample description.
		(b) Indicate number of participants with missing data for each variable of interest	Not applicable
		(c) Summarise follow-up time (eg, average and total amount)	Not applicable
Outcome data	15*	Report numbers of outcome events or summary measures over time	Subsection 3.2. General suicide rates by sex, methods and monthly trends
Main results	16	(*a*) Give unadjusted estimates and, if applicable, confounder-adjusted estimates and their precision (eg, 95% confidence interval). Make clear which confounders were adjusted for and why they were included	Subsections 3.3. Multivariate analysis of suicide mortality, 3.4. Age-specific suicide risk and 3.5. Impact of the COVID-19 pandemic.
		(*b*) Report category boundaries when continuous variables were categorized	Subsection 3.4. Age-specific suicide risk
		(*c*) If relevant, consider translating estimates of relative risk into absolute risk for a meaningful time period	IRR of subsections 3.2. to 3.5.
Other analyses	17	Report other analyses done—eg analyses of subgroups and interactions, and sensitivity analyses	Subsection 3.3. Multivariate analysis of suicide mortality
**Discussion**			
Key results	18	Summarise key results with reference to study objectives	Subsections 4.1. Spanish context and 4.2. Comparison with Japanese data
Limitations	19	Discuss limitations of the study, taking into account sources of potential bias or imprecision. Discuss both direction and magnitude of any potential bias	Subsection 4.4. Limitations.
Interpretation	20	Give a cautious overall interpretation of results considering objectives, limitations, multiplicity of analyses, results from similar studies, and other relevant evidence	Subsection 4.2. Comparison with Japanese data
Generalisability	21	Discuss the generalisability (external validity) of the study results	Subsection 4.3. Practical implications

**Other information**			
Funding	22	Give the source of funding and the role of the funders for the present study and, if applicable, for the original study on which the present article is based	Not applicable

*Give information separately for exposed and unexposed groups.

Note: An Explanation and Elaboration article discusses each checklist item and gives methodological background and published examples of transparent reporting. The STROBE checklist is best used in conjunction with this article (freely available on the Web sites of PLoS Medicine at http://www.plosmedicine.org/, Annals of Internal Medicine at http://www.annals.org/, and Epidemiology at http://www.epidem.com/). Information on the STROBE Initiative is available at http://www.strobe-statement.org.
